# Identifying New York City Neighborhoods at Risk of Being Overlooked for Interventions

**DOI:** 10.5888/pcd17.190325

**Published:** 2020-04-23

**Authors:** Joy X. Kang, Amber Levanon Seligson, Kacie L. Dragan

**Affiliations:** 1 NYC Department of Health and Mental Hygiene, New York, New York; 2 Harvard University Graduate School of Arts and Sciences, PhD Program in Health Policy, Cambridge, Massachusetts

## Abstract

Public health agencies are often faced with difficult decisions about where and how to allocate funding and resources. This question of resource allocation is central to public health policy; however, decisions related to resource allocation are sometimes made via informal or subjective approaches. We walk readers through a process of identifying needs across different neighborhoods in New York City (NYC) by examining community district–level health outcomes using data from published Community Health Profile reports released by the NYC Department of Health and Mental Hygiene (DOHMH) in 2015. In NYC, community districts are represented by community boards that provide a forum for addressing the needs of the community, making them a useful geographic unit for examining health information and turning data into action. We examined prevalence estimates and 95% confidence intervals of health indicators in each community district to identify significant disparities and calculated relative disparities in rates or prevalence to understand the relative magnitude of each disparity. Lastly, we demonstrate an application of this approach by identifying a cluster of neighborhoods with a high chance of being overlooked for public health interventions by conventional methods because of the relative number of disparities that exist in these neighborhoods. We present information on the disparity profile (number of disparities and relative disparity) for each neighborhood within the cluster and discuss potential public health implications. This approach can be applied to other jurisdictions to inform public health planning and resource allocation.

**Summary**

**What is already known about this topic?**

Public health agencies are frequently tasked with difficult decisions related to resource allocation. According to a 2011 survey of public health officials, fewer than half reported always or usually using structured means of making allocation decisions, despite most respondents saying needs assessments are moderately to very influential in decision making.

**What is added by this report?**

Using community-level health outcomes data for New York City (NYC), we assess the number of disparities each neighborhood has relative to its borough (county). We then apply this information to identify NYC neighborhoods that may be at risk of being overlooked for interventions because they represent a group with a moderate number of disparities.

**What are the implications for public health practice?**

This methodology for examining patterns of disparities that exist across neighborhoods is useful for public health planning and can aid in the making of efficient and equitable allocation decisions.

## Introduction

Public health agencies are often faced with difficult decisions about where to allocate funding and resources, including which initiatives to support and which ones to cut. This question of resource allocation is central to public health policy; however, decisions related to resource allocation are sometimes made via informal or subjective approaches (1). A national survey of local public health officials in 2011 found that fewer than half of all respondents usually or always used structured means of making allocation decisions (eg, economic analyses, conducting data-driven needs assessments) (1). However, 79% said needs assessments were moderately to very influential in making allocation decisions (1).

New York City comprises 5 boroughs (counties) and 59 community districts; socioeconomic and demographic characteristics and health disparities vary greatly across the city (2–5). The NYC Department of Health and Mental Hygiene (DOHMH) has a history of using a local approach to tackle public health problems at the neighborhood level. One example is the establishment of Neighborhood Health Action Centers (previously known as District Public Health Offices) in 2002 to promote health equity by targeting resources and programs in 3 high-need areas. More recently, in 2015 DOHMH compiled data on community characteristics and health outcomes at the community district level to create community health profiles (6). The community district geography was chosen for these reports because community districts are represented by community boards that provide a forum for addressing community needs, making them a useful geographic unit for examining health information and turning data into action. Community health profiles ranked community districts from best-performing to worst-performing on various indicators, with each profile providing information on that particular neighborhood relative to the city and relative to the borough in which it belonged. Community health profiles provided a resource for engaging community stakeholders in neighborhood planning and advocacy. For example, in 2016, DOHMH launched the Neighborhood Health Initiative in which 8 nonprofit organizations working in under-resourced neighborhoods were selected to bring community members together to review health data and to identify health equity goals (7). These organizations then partnered with community stakeholders to enact strategies to achieve these goals.

Neighborhood data, such as the community health profile, can be used to identify need within communities and also can be used as a resource in identifying need across communities to inform questions of allocation. The approach that we describe below can be used to identify neighborhoods with the highest need in a particular health domain or the neighborhood with the greatest number of different needs within a geographic unit. For this article, we used the health outcome data from community health profiles to identify neighborhoods with a high chance of being overlooked for interventions because they do not have a broad or sweeping range of health needs, but, rather, might benefit from targeted public health efforts in a small number of specific health areas. We defined these areas as those with a moderate (average or fewer than average) number of disparities on select health measures. The health measures chosen include a range of factors that were prioritized in NYC’s Take Care New York initiative (8), which have been shown to be associated with overall well-being, including health-related quality of life (9), reproductive health (10,11), substance use (12), mental health (13), interpersonal violence (14), chronic disease (15), and access to health care (16).

## Methods

### Identifying disparities

To identify neighborhoods at risk of being overlooked, we first compared the prevalence estimates and 95% confidence intervals (CIs) of each community district on 20 different health outcomes against the mean for the borough in which the community district belongs. We chose to use borough as the reference point because outcomes of interest tend to vary across boroughs, and using a citywide average would further mask the variation that occurs across NYC neighborhoods. The original community health profile reports include averages for community districts in addition to the citywide average and the average for the borough in which the community district is located (6).

We used health outcome data from the 2015 Community Health Profiles, which contained a total of 44 indicators (6). Prevalence estimates used in this article include multiple decimal places, whereas the public use data set (PUD) estimates were rounded to whole numbers or 1 decimal place. Because of differences in rounding, the PUD on the DOHMH website yields slightly different results than what is presented here (to obtain the data used for this article, email EpiDataRequest@health.nyc.gov). In addition, the measure of self-reported health used in this article was poor or fair health, whereas PUD indicated only excellent, very good, or good health. Similarly, we presented the percent of adults with no fruit or vegetable consumption in the last day, whereas the PUD indicator used was the percent of adults who reported eating at least 1 serving of fruits or vegetables per day. Lastly, we present an indicator of no exercise within the last 30 days, whereas the indicator reported in PUD is the percent of adults with any exercise in the last 30 days. We excluded indicators that did not directly measure health outcomes or access, such as the percent of people born outside of the United States or the rate of incarcerations. Although demographic, social, and economic indicators are important to consider in public health planning, our analysis focuses on mental and physical health outcomes and access to health care and therefore uses indicators directly measuring disease prevalence and health care access. The original community health profile indicators were chosen by a committee of researchers from bureaus within DOHMH, as well as department leadership, through a consensus process that considered the data quality of each potential indicator, its potential to contribute to comprehensive reports that joined together multiple indicators all in one place, its importance to population health, and how actionable it was.

We used the same method of comparison as was used for community health profiles to determine a significant disparity. When the 95% CI of the community district did not overlap with the CI for the borough and the point estimate was higher, we identified the community district as having a significant disparity relative to the borough. For each community district, this produced a list of health outcomes on which that community district was significantly worse off relative to the borough in which it was located.

### Defining cluster of interest

Community districts had a range of 0 to 17 health outcomes on which they had significant disparities relative to their borough, with a mean of 4 disparities. We used this mean number to define areas with few or moderate disparities (ie, areas with 1 to 4 disparities). We defined areas as having a greater-than-average number of disparities if they had more than 4 disparities when compared with the borough.

### Quantifying magnitude of disparities

To quantify the magnitude of each disparity, we calculated a percent of relative disparity. A relative disparity is the difference between a group and a reference point on a particular measure of health (17). We calculated the relative disparity by subtracting the borough prevalence rate from that of the community district, dividing this difference by the borough rate, and multiplying by 100:

(community district rate − borough rate)/(borough rate) × 100%

The resulting percent relative difference was then compared across community districts and across indicators. A relative disparity of 100% would indicate that the rate for the community district was twice as high as the rate for the borough.

## Results

Of 59 community districts, 23 had no difference in disparities when compared to borough averages. In other words, these community districts had CIs that overlapped with their boroughs on all outcomes. In contrast, 18 had a greater-than-average number of disparities relative to their borough. The number of disparities present in this greater-than-average group ranged from 5 to 17, with a mean of 9. The remaining 18 community districts fell into our primary group of interest, moderate disparities, which were those that had at least 1 but no more than 4 disparities. Most community districts that had moderate disparities were located in the boroughs of Queens and Brooklyn (Figure).

**Figure:**
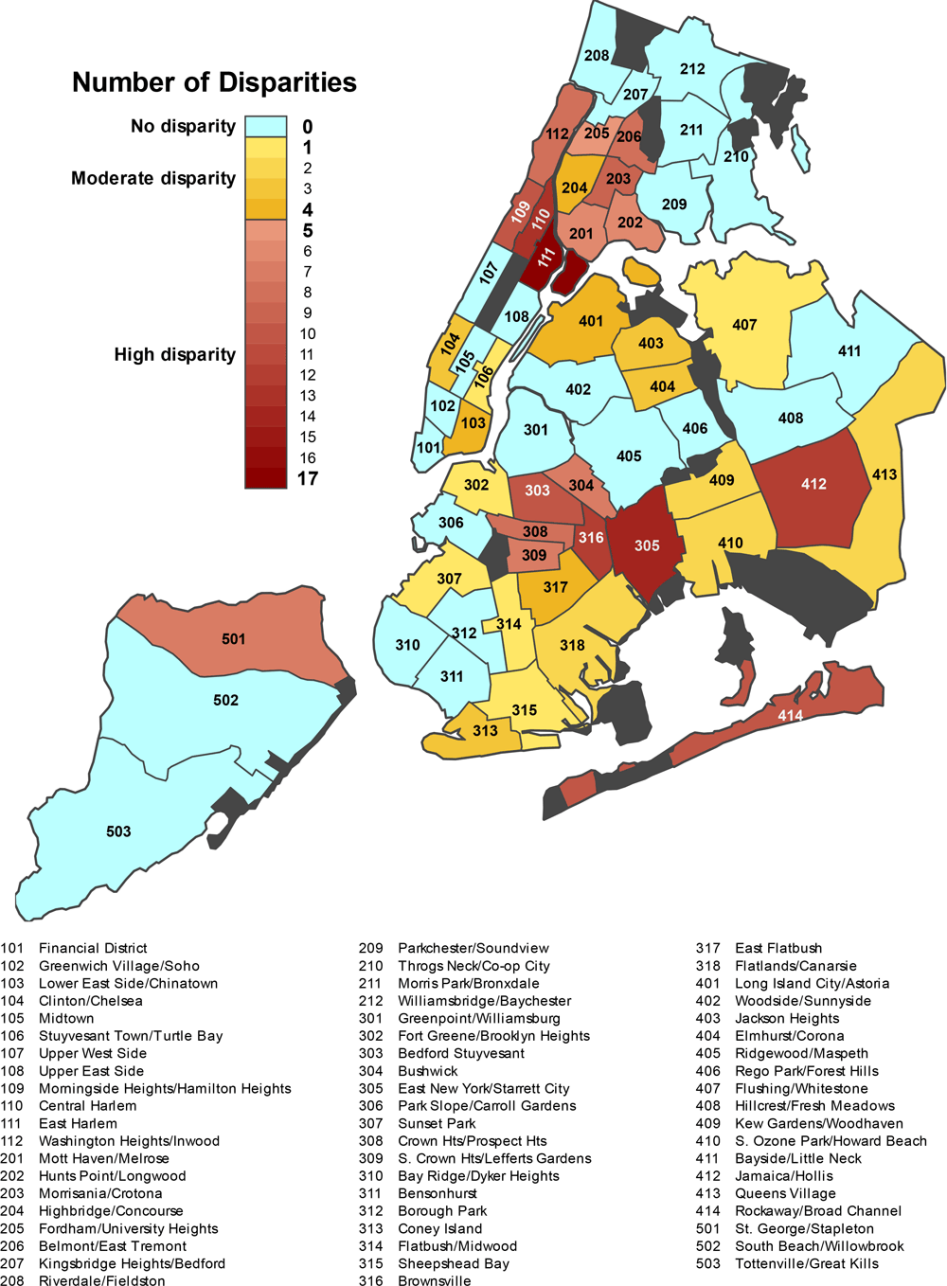
Map of New York City community districts by number of disparities relative to their borough. The map shows the 59 community districts and the disparity group of each district. Within the moderate and high disparity groups, darker colors indicate community districts with a greater number of health disparities.

**Table:** 

Community District Identification Number	Community District Name	Disparity Group	Number of Disparities
101	Financial District	None	0
102	Greenwich Village/Soho	None	0
105	Midtown	None	0
107	Upper West Side	None	0
108	Upper East Side	None	0
207	Kingsbridge Heights/Bedford	None	0
208	Riverdale/Fieldston	None	0
209	Parkchester/Soundview	None	0
210	Throgs Neck/Co-op City	None	0
211	Morris Park/Bronxdale	None	0
212	Williamsbridge/Baychester	None	0
301	Greenpoint/Williamsburg	None	0
306	Park Slope/Carroll Gardens	None	0
310	Bay Ridge/Dyker Heights	None	0
311	Bensonhurst	None	0
312	Borough Park	None	0
402	Woodside/Sunnyside	None	0
405	Ridgewood/Maspeth	None	0
406	Rego Park/Forest Hills	None	0
408	Hillcrest/Fresh Meadows	None	0
411	Bayside/Little Neck	None	0
502	South Beach/Willowbrook	None	0
503	Tottenville/Great Kills	None	0
106	Stuyvesant Town/Turtle Bay	Moderate	1
302	Fort Greene/Brooklyn Heights	Moderate	1
307	Sunset Park	Moderate	1
314	Flatbush/Midwood	Moderate	1
315	Sheepshead Bay	Moderate	1
407	Flushing/Whitestone	Moderate	1
318	Flatlands/Canarsie	Moderate	2
409	Kew Gardens/Woodhaven	Moderate	2
410	South Ozone Park/Howard Beach	Moderate	2
413	Queens Village	Moderate	2
104	Clinton/Chelsea	Moderate	3
313	Coney Island	Moderate	3
403	Jackson Heights	Moderate	3
404	Elmhurst/Corona	Moderate	3
103	Lower East Side/Chinatown	Moderate	4
204	Highbridge/Concourse	Moderate	4
317	East Flatbush	Moderate	4
401	Long Island City/Astoria	Moderate	4
205	Fordham/University Heights	High	5
201	Mott Haven/Melrose	High	6
202	Hunts Point/Longwood	High	6
304	Bushwick	High	7
309	South Crown Heights/Lefferts Gardens	High	7
501	St. George/Stapleton	High	7
112	Washington Heights/Inwood	High	8
206	Belmont/East Tremont	High	8
308	Crown Heights/Prospect Heights	High	8
203	Morrisania/Crotona	High	9
109	Morningside Heights/Hamilton Heights	High	10
303	Bedford Stuyvesant	High	10
414	Rockaway/Broad Channel	High	10
316	Brownsville	High	12
412	Jamaica/Hollis	High	12
110	Central Harlem	High	13
305	East New York/Starrett City	High	14
111	East Harlem	High	17

We listed all neighborhoods with moderate disparities along with the outcomes on which they were disparate and the relative disparity for each comparison (Table). The largest relative disparity at 132.8% was the outcome of late or no prenatal care in East Flatbush relative to Brooklyn as a whole. The smallest relative disparity was for drug hospitalizations in Clinton/Chelsea relative to Manhattan as a whole (9.1%). Numerous relative disparities surpassed 50%, indicating that these community districts had rates that were 50% worse than the rates in their respective boroughs. For example, the neighborhood of Jackson Heights had approximately 70% higher prevalence of residents reporting that they did not have health insurance compared with the overall prevalence of the uninsured in Queens.

**Table:** Relative Disparity and Total Number of Disparities^a^ for Community Districts With 1 to 4 Disparities Using Borough as the Reference, New York City, 2015

Relative Disparity,^b^ %														
Borough	CD^c^	1	2	3	4	5	6	7	8	9	10	11	12	No. of Disparities
**Manhattan**	103			73.5						35.2	18.1		76.2	4
	104	19.8	9.1		31.0									3
	106				18.1									1
**Brooklyn**	317						56.8	132.8	27.3		13.0			4
	313				26.3		28.4	71.9						3
	318						30.7	50.0						2
	302		11.9											1
	307					38.3								1
	315			46.4										1
	314							20.3						1
**Queens**	401	17.5	54.8						40.1	44.1				4
	403	17.3				73.8						69.7		3
	404			51.1		58.3						62.0		3
	409	21.2							35.6					2
	410	10.9									22.5			2
	413				37.6		33.3							2
	407			43.5										1
**Bronx**	204	10.3	15.1			26.7				17.5				4

Abbreviation: CD, community district.

a The table includes all neighborhoods with moderate disparities and the outcomes on which they were disparate, along with the relative disparity for each comparison. Health outcomes for which none of the community districts in our primary group of interest had a disparity relative to the borough are not shown.

b Numbers indicate the following: 1, alcohol hospitalizations (per 100,000); 2, drug hospitalizations (per 100,000); 3, poor/fair health; 4, psychiatric hospitalizations (per 100,000); 5, teen birth (per 1,000); 6, preterm birth (percent among live births); 7, late or no prenatal care (percent among live births); 8, nonfatal assault hospitalizations (per 100,000); 9, avoidable asthma hospitalizations (per 100,000); 10, avoidable diabetes hospitalizations (per 100,000); 11, no insurance; 12, diabetes.

c 103, Lower East Side/Chinatown; 104, Clinton/Chelsea; 106, Stuyvesant Town/Turtle Bay; 317, East Flatbush; 313, Coney Island; 318, Flatlands/Canarsie; 302, Fort Greene/Brooklyn Heights; 307, Sunset Park; 315, Sheepshead Bay; 314, Flatbush/Midwood; 401, Long Island City/Astoria; 403, Jackson Heights; 404, Elmhurst/Corona; 409, Kew Gardens/Woodhaven; 410, S. Ozone Park/Howard Beach; 413, Queens Village; 407, Flushing/Whitestone; 204, Highbridge/Concourse.

## Discussion and Public Health Implications

Neighborhoods with moderate disparities across multiple dimensions are likely to be overlooked in policy initiatives and interventions because they are not areas with the greatest number of disparities. However, these areas with specific unaddressed needs should be considered in allocation decisions. In fact, on the basis of magnitude, some of these areas had the largest relative disparity on particular outcomes. For example, compared with Brooklyn as a whole, East Flatbush had 4 disparities, including the largest relative disparity among all Brooklyn community districts for the percent of women with late or no prenatal care. This methodology for examining patterns of disparities that exist across neighborhoods is useful for public health planning and can help reach neighborhoods that may be at risk of being overlooked by flagging opportunities for targeted interventions in specific domains. These results can also inform resource allocation by highlighting areas that are geographically close to one another that may have common disparities and by potentially identifying patterns of disparities that co-exist within a geographical area. Our approach can also be modified so that clusters of interest are defined by the neighborhoods with the highest number of disparities or neighborhoods with the largest magnitude of disparities on specific health outcomes. In addition, our approach can be modified for less densely populated localities by conducting a county-level analysis in which counties are compared with the state, pending the availability of directly estimated data or modeled estimates (18–20). The flexibility of our approach lends itself to a multitude of applications to inform public health planning at the neighborhood level.

Our approach has several limitations. First, it relies on the availability of identical measures of health outcomes across all neighborhoods in a jurisdiction. Localities that do not have access to consistently collected data at small geographies would not be able to identify neighborhoods at risk of being overlooked by using this approach. Nevertheless, small area estimation is becoming more common (21,22), and localities could generate modeled estimates at geographic levels of interest. A second limitation is that formal statistical testing is not conducted in our approach, beyond comparison of CIs. An alternative would be to compare the community district to other community districts in the borough by using statistical hypothesis tests, but that would introduce the need for decisions about multiple comparison corrections, transforming a simple approach into one that would require more intensive calculations. Furthermore, the method we propose in this article keeps the emphasis on the absolute and relative magnitude of effect sizes relevant to policy makers’ allocation decisions rather than relying heavily on *P* values, which are highly sensitive to sample size and are easily misinterpreted or misused in policy decisions (23,24). A final limitation is that although the calculations described here are simple to conduct, they are time intensive if many geographic areas and multiple indicators need to be considered. Without an automated web- or software-based tool, this approach can be time-consuming. Limitations notwithstanding, in an era when the volume of data available to public health practitioners is continually growing, having approaches that facilitate strategic use of such data to inform efficient and equitable allocation decisions is increasingly important.
